# Impact of Sarcopenia on Percutaneous Epidural Balloon Neuroplasty in Patients with Lumbar Spinal Stenosis: A Retrospective Analysis

**DOI:** 10.3390/medicina59050847

**Published:** 2023-04-27

**Authors:** Yun-A Han, Hyun-Jung Kwon, Kunhee Lee, Min-Gi Son, Hotaek Kim, Seong-Soo Choi, Jin-Woo Shin, Doo-Hwan Kim

**Affiliations:** 1 Department of Anesthesiology and Pain Medicine, National Police Hospital, Seoul 05715, Republic of Korea; yyyuna@police.go.kr (Y.-A.H.); hipnotizer@police.go.kr (H.K.); 2 Department of Anesthesiology and Pain Medicine, Asan Medical Center, University of Ulsan College of Medicine, Seoul 05505, Republic of Korea; kwonhj@amc.seoul.kr (H.-J.K.); kunhee2727@gmail.com (K.L.); genesis1007@naver.com (M.-G.S.); choiss@amc.seoul.kr (S.-S.C.); sjinwoo@hotmail.com (J.-W.S.)

**Keywords:** sarcopenia, chronic pain, balloon, epidural, lumbar, neuroplasty, percutaneous, spinal stenosis

## Abstract

*Background and Objectives*: With the aging population, the incidence of degenerative lumbar spinal stenosis (LSS) is increasing. Sarcopenia is an age-related muscular decrease. Although epidural balloon neuroplasty is effective in patients with LSS refractory to conventional treatments, its effect has not been assessed in patients with sarcopenia. Therefore, this study evaluated the effect of epidural balloon neuroplasty in patients with LSS and sarcopenia. *Materials and Methods*: This retrospective study reviewed the following details from the electronic medical records: patient characteristics, including sex, age, body mass index, diabetes, hypertension, stenosis grading, pain duration, location, pain intensity, and medications. Back and leg pain intensity was evaluated before and after the procedure at one, three, and six months during the follow-up period. A generalized estimating equations model was used at six months follow-up. Patients were divided into sarcopenia and non-sarcopenia groups using the cross-sectional area of the psoas muscle at the level of L3 on magnetic resonance imaging. *Results*: A total of 477 patients were included (sarcopenia group: 314 patients, 65.8%; non-sarcopenia group: 163 patients, 34.2%). Age, sex, body mass index, and medication quantification scale III were statistically different between both groups. The generalized estimating equations analyses—with unadjusted and adjusted estimation—revealed a significantly reduced pain intensity after the procedure compared to the baseline in both groups. The difference in pain intensity between both groups was not statistically different. *Conclusions*: Percutaneous epidural balloon neuroplasty may be considered for patients with chronic lumbar LSS regardless of accompanying sarcopenia.

## 1. Introduction

The aging process is accompanied by changes in body composition, with loss of skeletal muscle and lean body mass, reduced bone mineral density, and increased fat mass. The European Working Group on Sarcopenia in Older People (EWGSOP) defined sarcopenia as a progressive and generalized loss of skeletal muscle mass and muscle strength or physical performance with advancing age [1]. Sarcopenia, the age-related loss of muscle mass and function, is a multifactorial condition influenced by a variety of mechanisms [2]. One key mechanism is changes in muscle fiber number and cross-sectional area of the remaining muscle fibers. With advancing age, individuals may undergo a reduction in the overall quantity of muscle fibers as well as a decrease in the size of individual muscle fibers, ultimately resulting in muscle atrophy. Another important factor is altered protein metabolism, where there is an imbalance between protein synthesis and protein degradation. This imbalance can result in a net loss of muscle mass over time. Hormonal changes also play a role in sarcopenia. There is evidence that levels of growth hormone and testosterone, which are important for muscle growth and maintenance, decrease with age. On the other hand, there may be an increase in cortisol, a stress hormone, and cytokines, which are markers of inflammation, both of which can contribute to muscle loss. Furthermore, changes in gene expression and cellular processes, such as apoptosis, can impact muscle health and contribute to sarcopenia [2,3]. These complex mechanisms interact with each other and can collectively contribute to the development and progression of sarcopenia.

Sarcopenia is associated with a risk of adverse outcomes, such as physical disability, poor quality of life, and death [1,4]. Recently, there have been several reports that sarcopenia is associated with adverse postoperative outcomes, increased morbidity, and mortalities in several medical conditions [3,5,6,7,8,9]. Therefore, appropriate evaluation of sarcopenia is emphasized to predict postoperative outcomes in older adults [10].

Most older adults experience low back pain, leg pain, and claudication associated with lumbar spinal stenosis (LSS) [11,12,13,14]. Elderly patients with LSS have a high prevalence of sarcopenia [15,16]; these cases are often refractory to conventional management, such as medications, physical therapy, lifestyle modification, and epidural steroid injections [12]. Percutaneous epidural balloon neuroplasty (PEBN) combines balloon decompression and conventional epidural neuroplasty, yielding significant pain reduction and improvement of functional capacity in patients with LSS [17,18]. Notably, these improvements provided long-term benefits up to 12 months after the procedure [19]. Several studies have demonstrated that PEBN can effectively treat LSS that is refractory to conventional management [17,18,19,20].

Although sarcopenia is known to be associated with poor prognosis after lumbar spine surgeries [21,22,23], only a few studies exist on sarcopenia’s effect in epidural procedures [24]. In addition, there is no study on the effect of sarcopenia in patients with LSS who underwent PEBN. Therefore, in this longitudinal cohort study, we assessed the impact of sarcopenia in patients with LSS who underwent PEBN.

## 2. Materials and Methods

### 2.1. Study Design and Participants 

This study was retrospectively conducted at our pain clinic. All data used in this study were itemized and filled out in the patients’ electronic medical records (EMR) when the patient visited the pain clinic. Our institutional review board (IRB number, 2019-1612) approved this retrospective study and waived the requirement for obtaining informed consent because only documented data were reviewed.

We reviewed the data of all patients who had undergone PEBN from January 2014 to December 2018. The inclusion criteria were (1) patients aged ≥ 65 years with radicular leg pain and/or back pain; (2) patient’s symptoms have persisted for over three months; (3) lumbar central or foraminal stenosis (any grade) was diagnosed using magnetic resonance imaging (MRI); (4) conventional treatment, including typical epidural steroid injections, had failed. The exclusion criteria were as follows: (1) history of prior lumbar spine surgery before balloon neuroplasty, (2) patients without proper radiologic images (MRI), (3) those who had no available data in EMR, (4) patients lost to follow-up entirely after the procedure.

### 2.2. Percutaneous Epidural Balloon Neuroplasty

The procedure started with meticulous sterile preparation to ensure aseptic conditions. The patient’s skin and soft tissues were carefully infiltrated with 1% lidocaine for local anesthesia. To access the epidural space, a specially designed 10-G guide needle was inserted through the sacral hiatus under intermittent fluoroscopy, taking care to prevent cutting or skiving of the catheter.

To confirm the correct placement of the guide needle in the epidural space, approximately 8 mL of diluted contrast medium (Omnipaque; Nycomed Imaging AS, Oslo, Norway) was injected under fluoroscopy. The diluted contrast mixture consisted of approximately 4 mL of pure contrast medium, 4 mL of 1% lidocaine, and 1500 IU of hyaluronidase. The contrast flow was carefully observed to check for any filling defects, which could indicate potential complications such as intravascular placement of the needle or contrast. If any issues were identified, the needle was promptly repositioned to ensure accurate placement.

Once the epidurogram and target areas were appropriately identified, an epidural inflatable balloon catheter (ZiNeu^®^, JUVENUI, Seoul, Republic of Korea) was advanced through the guide needle to the site of the filling defect or the pathology site, as determined by MRI or symptomatology. Gentle mechanical adhesiolysis and decompression were then performed using the ZiNeu catheter at the designated target sites, which included the central ventral and dorsal epidural spaces, lateral recess area, and/or each intervertebral foramen.

The adhesiolysis and decompression were carried out using a gentle side-to-side movement of the catheter with intermittent ballooning. The balloon was inflated with 0.13 mL of contrast agent using a 1 mL Luer-Lock syringe (BD Medical, Franklin Lakes, NJ, USA), and each ballooning process was limited to five seconds to minimize the risk of adverse effects. The degree of balloon inflation was adjusted based on the patient’s pain response; if moderate-to-severe pain was reported during balloon inflation, no further attempts were made due to safety concerns. It is important to note that the catheter remained deflated while being moved to avoid any potential complications.

After adhesiolysis and decompression, 1 mL of pure contrast medium was injected to detect subarachnoid or intravascular filling and to ensure satisfactory filling of previous defects. Subsequently, 2 mL of 1% lidocaine combined with 5 mg of dexamethasone was injected at each target site to provide pain relief and reduce inflammation. At the conclusion of the procedure, a Perifix epidural catheter (B. Braun Melsungen AG, Melsungen, Germany) was left in place at the main target site through the ZiNeu catheter lumen. The position of the Perifix catheter tip was confirmed, and the ZiNeu catheter was then carefully removed.

In the recovery room, a test injection of 2 mL of lidocaine was administered via the Perifix catheter. After monitoring for 10–15 minutes to ensure there were no adverse reactions, another 4 mL of 10% hypertonic saline was injected via the Perifix catheter. The Perifix catheter was left in place for a two-day drug injection regimen.

On the second day post-procedure, the Perifix catheter was removed after injecting the same drugs (2 mL of 1% lidocaine and 4 mL of 10% hypertonic saline with 5 mg of dexamethasone) following another test injection. All patients were discharged after confirming.

### 2.3. Demographic Data and Outcome Assessments

Baseline characteristics, including age, sex, body mass index (BMI), hypertension, diabetes, pain duration, location, intensity, and medications, were retrieved from the EMR. The location, grade, and total number of LSS (central and foraminal) were recorded from MRI images using a photo archiving and communication system (PetaVision, Version 2.1, Seoul, Republic of Korea).

The measurement of the cross-sectional muscle area of the psoas muscle was conducted using T2 weighted axial images of MRI at the L3 level, as outlined in a previous study [25]. The fascial boundary of the left and right psoas and multifidus muscles was manually outlined, and the resulting area was normalized by the square of body height to obtain a measurement in cm^2^/m^2^ [6]. Sarcopenia was defined based on established thresholds of total psoas area, with values of 564.2 mm^2^/m^2^ for men and 414.5 mm^2^/m^2^ for women at the L3 level [25]. This determination of sarcopenia was used to identify accompanying sarcopenia in patients with lumbar spinal stenosis (LSS) in the present study.

The primary outcomes of this study were assessed based on improved pain intensity in the back and leg, as measured using an 11-point numeric rating scale (NRS). In addition, the medication quantification scale III (MQS) was utilized to quantify analgesic changes, and these assessments were conducted at the baseline, as well as at one, three, and six months after the procedure [26]. These outcomes were individually recorded during the follow-up period. To evaluate the impact of sarcopenia on the outcomes of the procedure, patients with LSS were divided into two groups: sarcopenia and non-sarcopenia groups. The severity of central spinal stenosis was graded as mild, moderate, or severe based on the cerebrospinal fluid/rootlet ratio observed in axial T2 MRI images. The assessment of stenosis was based on the ratio of CSF to rootlets observed on axial T2 images. The grading system was as follows: mild stenosis indicated that there was CSF visible within the dural sac, but its distribution was uneven. Moderate stenosis indicated that the rootlets occupied the entire dural sac—but they were still distinguishable and there was some CSF present—resulting in a grainy appearance of the sac. Severe stenosis denoted that the dural sac appeared homogeneous with a grey signal, and no rootlets were recognizable, while epidural fat was present posteriorly or not recognizable [27]. Similarly, foraminal stenosis was graded as mild, moderate, or severe based on the degree of horizontal and vertical stenosis of the nerve root from the surrounding disc, facet joint, or ligamentum flavum, as seen in the MRI sagittal view. Mild foraminal stenosis indicated perineural fat obliteration encircling the nerve root in either the vertical or transverse directions. This involved contact with the superior and inferior or anterior and posterior regions of the nerve root, without any visible morphological changes. Moderate foraminal stenosis corresponded to perineural fat obliteration encompassing the nerve root in all four directions, without any observable morphological changes in both the vertical and transverse planes. Severe foraminal stenosis denoted nerve root collapse or morphological changes [28]. These assessments were conducted to provide a comprehensive evaluation of the impact of sarcopenia on the outcomes of the procedure in patients with LSS.

### 2.4. Statistical Analysis

Continuous variables were shown as the median and interquartile range or mean and standard deviation if skewed. Frequencies and percentages were used to present categorical variables. To compare the baseline differences between the two groups, we compared continuous variables using the Student’s *t*-test or Mann–Whitney U test, as appropriate. We used Fisher’s exact tests or Pearson’s chi-square to analyze the categorical variables between the two groups.

We used a generalized estimating equations (GEE) model to replace missing data throughout the follow-up period. Stata version 13.1 (StataCorp LP, College Station, TX, USA) and IBM SPSS Statistics for Windows, version 22.0 (IBM Corp., Armonk, NY, USA), were conducted for data manipulation and statistical analysis. A value of *p* < 0.05 was statistically significant.

## 3. Results

**Figure 1:**
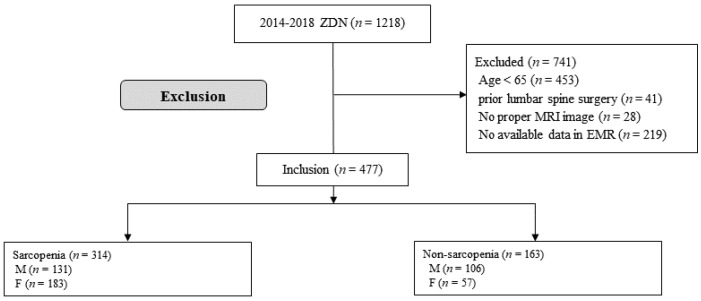
Study flow chart.

During the study period, 1218 patients who underwent PEBN were enrolled in the entire cohort; 741 were excluded based on the study criteria. Among the 741 excluded patients, 453 were younger than 65 years, 41 had undergone lumbar spine surgery before PEBN, 28 had no or poor radiologic images, and 219 had non-available clinical data. Eventually, this study included 477 patients who were divided into the sarcopenia (314 patients, 65.8%) and non-sarcopenia (163 patients, 35.2%) groups (Figure 1).

Table 1 shows the baseline and intervention characteristics of the study population. The median NRS of pain intensity was 7.0 (6.0–8.0) for both back and leg pain. The median values of MQS and pain duration of the total study population were 8.2 and 24 months, respectively. The patients in the sarcopenia group were older than the non-sarcopenia group (73.0 (69.0–78.0) vs. 71.0 (68.0–77.0) years, *p* = 0.031). The non-sarcopenia group had a significantly higher proportion of male patients (65% vs. 41.7%, *p* = 0.001) and a significantly higher BMI (25.8 (23.9–28.0) vs. 24.1 (22.5–26.0) kg/m^2^, *p* = 0.001). Additionally, preoperative MQS in the sarcopenia group was significantly lower than that in the non-sarcopenia group (6.8 (4.2–11.6) vs. 9.6 (4.6–13.8), *p* = 0.002). Table 2 shows the unadjusted estimation of values and differences between both groups from the baseline for the NRS score of back pain and leg pain over the six-month follow-up period. In both groups, GEE analyses revealed a significant improvement in the estimated mean NRS at one, three, and six months after the procedure compared to the baseline (*p* < 0.001). The estimated mean NRS of back and leg pain did not differ significantly between the groups throughout the study period.

Table 3 shows the adjusted estimation of values and differences between the groups from the baseline for the NRS of leg and back pain over the six-month follow-up period. GEE analysis revealed a significant reduction in the estimated mean NRS compared to the baseline throughout the follow-up period in both groups (*p* < 0.001). When comparing both groups using a GEE model, there were no significant differences in the NRS for leg and back pain. The *p*-values of the interactions between the groups and times for back pain and leg pain were 0.347 and 0.684, respectively.

## 4. Discussion

To our knowledge, no studies have reported the impact of sarcopenia on PEBN in patients with LSS. Our results revealed that PEBN significantly decreased pain intensity up to six months after the procedure in patients with LSS regardless of sarcopenia.

Due to an aging population, aging is now receiving increased social and medical attention. In elderly patients, frailty, such as weight loss or lack of physical activity, is a significant risk for geriatric diseases [29]. In 1989, Rosenberg et al. first defined sarcopenia as a progressive loss of muscle mass with advancing age [30]. According to EWGSOP, the prevalence of sarcopenia in individuals between 60 and 70 years of age is 5–13%, while its prevalence in those older than 80 years ranges from 11–50% [1,4].

Various studies on sarcopenia have recently been conducted due to an increase in the elderly population. The presence of sarcopenia is associated with poor outcomes after surgery [21,22,31], increasing mortality in patients in the intensive care unit, and increasing the incidence of hospitalization and length of hospital stay [32].

There were some limitations in diagnosing sarcopenia using the CSA of the psoas muscle. However, several studies demonstrated that reduced CSA of the psoas muscle measured through CT or MRI may represent sarcopenia [3,25]. Lower psoas muscle CSA has been associated with increased mortality [33,34] and higher risks in patients undergoing cardiac operations [35], suggesting that psoas CSA may be a useful predictor of clinical outcomes.

LSS is known to be more prevalent in older individuals, as it is often associated with degenerative changes in the spine that occur with aging [13]. The treatment of LSS includes conservative measures such as physical therapy, pain management, and exercise, as well as more invasive options like epidural steroid injections, surgery, or PEBN depending on symptom severity and patient health [12,17,18,36].

PEBN is a safe and effective treatment for intractable lumbar radicular and back pain and functional improvements [17,18,19,20]. It is thought to decompress the epidural space and lyse the adhesion more effectively than conventional neuroplasty without an inflatable catheter [17,18]. The effectiveness of PEBN compared to conventional neuroplasty may be explained by several factors. First, intermittent ballooning of the epidural space may lead to more effective mechanical detachment of perineural adhesion, which can alleviate symptoms and improve function [18]. Second, PEBN may be more easily and effectively performed on target lesions, as the ZiNeu catheter can be manipulated vertically and horizontally. Therefore, epidural drugs such as local anesthetics, steroids, and hypertonic saline can be more successfully delivered to the target lesion [18]. Additionally, after ballooning, there is an advantage in administering drugs continuously with a time interval by inserting a catheter. Third, the combined mechanical balloon decompression and adhesiolysis may reduce venous congestion, possibly causing a circulatory disturbance and inducing neurogenic claudication [37].

Redundant nerve root and degenerative lumbar spondylolisthesis are generally associated with poor outcomes in surgery or pain procedures [17,38,39]. However, recent studies on the evaluation of predictive factors of PEBN showed that it provided long-term pain relief regardless of the redundant nerve root or degenerative lumbar spondylolisthesis in patients with LSS [17,18]. These results were in line with the results of the present study. The advantages of PEBN mentioned above may have contributed to the long-term effects of PEBN in patients with sarcopenia.

Regarding demographic data, the BMI, age, sex, and MQS were statistically different between both groups. Several reports show that older adults tend to have low BMIs, which can cause muscle loss and bone density problems [29,40]. Patients with sarcopenia were generally older and had a lower BMI. Additionally, compared to male patients, a higher prevalence of sarcopenia was observed among female patients. This could be because body composition differs between men and women, with women having proportionally more fat mass and men having proportionally more muscle mass [41,42]. The baseline MQS was higher in the non-sarcopenia group than in the sarcopenia group. Due to the younger age and higher BMI in the non-sarcopenia group, more analgesics might have been prescribed for a similar pain intensity.

Our study had several limitations. Firstly, physical functions measured via objective exams, such as gait speed and muscle strength, were not included in the evaluation of sarcopenia [3]. However, many studies have defined sarcopenia through image analysis. Since it is difficult to check physical function in clinical practice, image-based analysis of sarcopenia is considered meaningful [3,25]. Secondly, we only measured the CSA and did not consider the fat infiltration of the psoas muscles. Changes in muscle function and quality have been emphasized in the diagnosis of sarcopenia recently [24]. Therefore, further studies about the impact of fat infiltration on PEBN are needed. Thirdly, we did not evaluate physical functional status improvement after PEBN. However, evaluating NRS and MQS for one, three, and six months is considered an appropriate method of estimating the patient’s symptom relief. Finally, the retrospective study could have possibly reported undocumented factors or biases. However, by using GEE to adjust for variables, we tried to minimize the impact of confounding factors that could have affected the outcome. Therefore, a well designed randomized controlled study is necessary to evaluate the outcomes of this therapeutic approach in the future.

## 5. Conclusions

PEBN may lead to considerable pain reduction for at least six months in patients with chronic LSS accompanying sarcopenia. PEBN might be considered for patients with chronic LSS regardless of sarcopenia being refractory to conservative treatments.

## Figures and Tables

**Table 1:** Baseline characteristics of the patients.

Variables	Total (*n* = 477)	Sarcopenia		*p*-Value
		No (*n* = 163)	Yes (*n* = 314)	
Age, year	72.0 (69.0–77.0)	71.0 (68.0–77.0)	73.0 (69.0–78.0)	0.031
Sex, male	237 (49.7)	106 (65.0)	131 (41.7)	0.001
BMI, kg/m^2^	24.8 (22.9–26.6)	25.8 (23.9–28.0)	24.1 (22.5–26.0)	0.001
Diabetes	113 (23.7)	35 (21.5)	78 (24.8)	0.480
Hypertension	240 (50.3)	82 (50.3)	158 (50.3)	0.999
Pain duration, months	24.0 (12.0–48.0)	26.0 (12.0–62.0)	24.0 (12.0–48.0)	0.058
Pain location				
Back/leg/both	42 (8.8)/141 (29.6)/294 (61.6)	10 (6.1)/48 (29.4)/105 (64.4)	32 (10.2)/93 (29.6)/189 (60.2)	0.314
Pain intensity (NRS)				
Back pain	7.0 (6.0–8.0)	7.0 (6.0–8.0)	7.0 (6.0–8.0)	0.844
Leg pain	7.0 (6.0–8.0)	7.0 (6.0–8.0)	7.0 (6.0–8.0)	0.565
MQS	8.2 (4.2–12.4)	9.6 (4.6–13.8)	6.8 (4.2–11.6)	0.002
Central stenosis grading				
Mild/Moderate/ Severe	117 (25.0)/98 (20.9)/253 (53.8)	31 (19.5)/30 (18.9)/ 98 (61.6)	86 (27.8)/68 (22.0)/155 (50.3)	0.093
Foraminal stenosis grading				
Mild/Moderate /Severe	139 (33.4)/123 (29.6)/154 (37.0)	55 (37.9)/44 (30.3)/ 46 (31.7)	84 (31.0)/79 (29.2)/108 (39.9)	0.213
Target level				0.179
1 level	208 (43.6)	69 (42.3)	139 (44.3)	
2 levels	191 (40.0)	65 (39.9)	126 (40.1)	
3 levels	67 (14.0)	28 (17.2)	39 (12.4)	
>4 levels	11 (2.3)	1 (0.6)	10 (3.2)	

Values are expressed as means ± standard deviations, medians (interquartile ranges), or numbers (percentages). BMI, body mass index; MQS, medication quantification scale.

**Table 2:** Changes in the estimated pain scores and medication quantification scores after percutaneous balloon neuroplasty in patients with chronic spinal stenosis with and without sarcopenia.

Variables	Time	Estimated Pain Score (95% CI)		Estimated Difference (95% CI) *	*p*-Value
		Non-Sarcopenia (*n* = 163)	Sarcopenia (*n* = 314)		
Back pain (NRS)	Baseline	6.7 (6.3–7.1)	6.7 (6.5–7.0)	0.0 (−0.5–0.5)	0.912
	1 month	4.9 (4.5–5.3) ^†^	4.7 (4.4–5.0) ^†^	−0.2 (−0.7–0.3)	0.463
	3 months	4.9 (4.4–5.5) ^†^	4.6 (4.2–4.9) ^†^	−0.4 (−1.0–0.3)	0.262
	6 months	4.8 (4.2–5.5) ^†^	4.4 (4.0–4.9) ^†^	−0.4 (−1.2–0.4)	0.343
Leg pain (NRS)	Baseline	7.0 (6.7–7.4)	7.2 (6.9–7.4)	0.2 (−0.3–0.6)	0.456
	1 month	5.2 (4.8–5.5) ^†^	4.9 (4.6–5.2) ^†^	−0.3 (−0.7–0.2)	0.241
	3 months	4.6 (4.1–5.0) ^†^	4.7 (4.3–5.0) ^†^	0.1 (−0.5–0.6)	0.758
	6 months	4.5 (4.0–5.0) ^†^	4.7 (4.3–5.1) ^†^	0.2 (−0.5–0.9)	0.540
MQS	Baseline	9.6 (8.6–10.6)	7.6 (6.8–8.4)	−2.0 (−3.2–0.7)	0.003
	1 month	9.7 (8.6–10.7)	8.2 (7.4–8.9)	−1.5 (−2.8–0.3)	0.015
	3 months	9.7 (8.6–10.9)	8.2 (7.4–9.0)	−1.6 (−2.9–0.2)	0.028
	6 months	8.7 (7.4–9.9)	7.4 (6.5–8.3)	−1.3 (−2.8–0.3)	0.111

A numerical rating scale (NRS) was used to assess lower back and leg pain intensity. A generalized estimating equation model was used in the statistical analysis. * Estimated difference in values between groups. ^†^ *p* < 0.001 compared to the baseline in each group. MQS, medication quantification scale.

**Table 3:** Adjusted changes in the estimated pain scores and medication quantification scores after percutaneous balloon neuroplasty in patients with chronic spinal stenosis with and without sarcopenia.

Variables	Time	Estimated Pain Score (95% CI)		Estimated Difference (95% CI) *	*p*-Value
		Non-Sarcopenia (*n* = 163)	Sarcopenia (*n* = 314)		
Back pain (NRS)	Baseline	7.1 (6.6–7.6)	6.9 (6.6–7.3)	−0.1 (−0.8–0.5)	0.685
	1 month	5.2 (4.5–5.7) ^†^	4.8 (4.4–5.2) ^†^	−0.4 (−1.0–0.3)	0.257
	3 months	5.3 (4.5–6.0) ^†^	4.8 (4.3–5.2) ^†^	−0.5 (−1.4–0.4)	0.268
	6 months	5.1 (4.2–6.0) ^†^	4.6 (3.9–5.3) ^†^	−0.5 (−1.7–0.6)	0.347
Leg pain (NRS)	Baseline	7.3 (6.9–7.7)	7.4 (7.0–7.7)	0.1 (−0.5–0.6)	0.846
	1 month	5.4 (5.0–5.8) ^†^	5.0 (4.7–5.3) ^†^	−0.4 (−1.0–0.1)	0.145
	3 months	4.5 (3.8–5.1) ^†^	4.8 (4.4–5.2) ^†^	0.3 (−0.4–1.1)	0.407
	6 months	4.9 (4.1–5.6) ^†^	5.1 (4.4–5.7) ^†^	0.2 (−0.8–1.2)	0.684
MQS	Baseline	10.1 (8.9–11.3)	7.8 (6.9–8.7)	−2.3 (−3.8–0.8)	0.003
	1 month	9.7 (8.6–10.9)	8.4 (7.6–9.2)	−1.3 (−2.8–0.1)	0.071
	3 months	9.7 (8.4–10.9)	8.4 (7.5–9.3)	−1.3 (−2.9–0.3)	0.112
	6 months	9.1 (7.6–10.5)	7.6 (6.6–8.6)	−1.4 (−3.2–0.3)	0.114

A numerical rating scale (NRS) was used to assess lower back and leg pain intensity. A generalized estimating equation model was used in the statistical analysis. Age, sex, body mass index, and medication quantification scale were included to adjust for demographic differences. Data are presented as estimated means with 95% confidence intervals (CIs). * Estimated difference in values between groups. ^†^ *p* < 0.001 compared to the baseline in each group. MQS, medication quantification scale.

## Data Availability

Data is available upon reasonable request.

## References

[1] Cruz-Jentoft A.J., Baeyens J.P., Bauer J.M., Boirie Y., Cederholm T., Landi F., Martin F.C., Michel J.-P., Rolland Y., Schneider S.M. (2010). Sarcopenia: European consensus on definition and diagnosisReport of the European Working Group on Sarcopenia in Older People. Age Ageing.

[2] Doherty T.J. (2003). Invited review: Aging and sarcopenia. J. Appl. Physiol..

[3] Bentov I., Kaplan S.J., Pham T.N., Reed M.J. (2019). Frailty assessment: From clinical to radiological tools. Br. J. Anaesth..

[4] Campins L., Camps M., Riera A., Pleguezuelos E., Yebenes J.C., Serra-Prat M. (2017). Oral drugs related with muscle wasting and sarcopenia. A review. Pharmacology.

[5] Alipour O., Lee V., Tejura T.K., Wilson M.L., Memel Z., Cho J., Cologne K., Hwang C., Shao L. (2021). The assessment of sarcopenia using psoas muscle thickness per height is not predictive of post-operative complications in IBD. Scand. J. Gastroenterol..

[6] Zager Y., Khalilieh S., Ganaiem O., Gorgov E., Horesh N., Anteby R., Kopylov U., Jacoby H., Dreznik Y., Dori A. (2021). Low psoas muscle area is associated with postoperative complications in Crohn’s disease. Int. J. Colorectal Dis..

[7] Nakashima Y., Saeki H., Nakanishi R., Sugiyama M., Kurashige J., Oki E., Maehara Y. (2018). Assessment of sarcopenia as a predictor of poor outcomes after esophagectomy in elderly patients with esophageal cancer. Annal. Sur..

[8] Hsu J., Krishnan A., Lin C.T., Shah P.D., Broderick S.R., Higgins R.S., Merlo C.A., Bush E.L. (2019). Sarcopenia of the psoas muscles is associated with poor outcomes following lung transplantation. Annal. Thorac. Sur..

[9] Druckmann I., Yashar H., Schwartz D., Schwartz I.F., Goykhman Y., Ben-Bassat O.K., Baruch R., Tzadok R., Shashar M., Cohen-Hagai K. (2022). Presence of Sarcopenia before Kidney Transplantation Is Associated with Poor Outcomes. Am. J. Nephrol..

[10] Nakai Y., Makizako H., Kiyama R., Tomioka K., Taniguchi Y., Kubozono T., Takenaka T., Ohishi M. (2019). Association between chronic pain and physical frailty in community-dwelling older adults. Int. J. Environ. Res. Public Health.

[11] Deer T., Sayed D., Michels J., Josephson Y., Li S., Calodney A.K. (2019). A review of lumbar spinal stenosis with intermittent neurogenic claudication: Disease and diagnosis. Pain Med..

[12] Lurie J., Tomkins-Lane C. (2016). Management of lumbar spinal stenosis. BMJ.

[13] Szpalski M., Gunzburg R. (2003). Lumbar spinal stenosis in the elderly: An overview. Eur. Spine J..

[14] Kaptan H., Kasimcan O., Cakiroglu K., Ilhan M.N., Kilic C. (2007). Lumbar spinal stenosis in elderly patients. Annal. N. Y. Acad. Sci..

[15] Park S., Kim H., Ko B., Chung J., Kim S., Park S., Lee M., Yeom J. (2016). The prevalence and impact of sarcopenia on degenerative lumbar spinal stenosis. Bone Joint J..

[16] Sakai Y., Matsui H., Ito S., Hida T., Ito K., Koshimizu H., Harada A. (2017). Sarcopenia in elderly patients with chronic low back pain. Osteoporos. Sarcopenia.

[17] Karm M.-H., Kim C.-S., Kim D.-H., Lee D., Kim Y., Shin J.-W., Choi S.-S. (2023). Effectiveness of percutaneous epidural neuroplasty using a balloon catheter in patients with chronic spinal stenosis accompanying mild spondylolisthesis: A longitudinal cohort study. Korean J. Pain.

[18] Karm M.-H. (2022). Effectiveness of epidural balloon neuroplasty in patients with chronic spinal stenosis accompanied by redundant nerve roots: A longitudinal cohort study. Pain Physician.

[19] Kim N.E., Choi J.B., Kwon H.R., Chung H.T., Jang E.S., Moon C.Y., Kim B.G. (2022). Comparison of the Effect of Balloon Catheter vs Nucleoplasty vs Balloon Catheter and Nucleoplasty in Patients With Lumbar Spinal Stenosis. Pain Physician.

[20] Gil H.Y., Jeong S., Cho H., Choi E., Nahm F.S., Lee P.-B. (2019). Kambin’s triangle approach versus traditional safe triangle approach for percutaneous transforaminal epidural adhesiolysis using an inflatable balloon catheter: A pilot study. J. Clin. Med..

[21] Bokshan S.L., Han A.L., DePasse J.M., Eltorai A.E., Marcaccio S.E., Palumbo M.A., Daniels A.H. (2016). Effect of sarcopenia on postoperative morbidity and mortality after thoracolumbar spine surgery. Orthopedics.

[22] Hirase T., Haghshenas V., Bratescu R., Dong D., Kuo P.H., Rashid A., Kavuri V., Hanson D.S., Meyer B.C., Marco R.A. (2021). Sarcopenia predicts perioperative adverse events following complex revision surgery for the thoracolumbar spine. Spine J..

[23] Inose H., Yamada T., Hirai T., Yoshii T., Abe Y., Okawa A. (2018). The impact of sarcopenia on the results of lumbar spinal surgery. Osteoporos. Sarcopenia.

[24] Kim H.J., Rho M., Yoon K.B., Jo M., Lee D.W., Kim S.H. (2022). Influence of cross-sectional area and fat infiltration of paraspinal muscles on analgesic efficacy of epidural steroid injection in elderly patients. Pain Pract..

[25] Amini N., Spolverato G., Gupta R., Margonis G.A., Kim Y., Wagner D., Rezaee N., Weiss M.J., Wolfgang C.L., Makary M.M. (2015). Impact total psoas volume on short-and long-term outcomes in patients undergoing curative resection for pancreatic adenocarcinoma: A new tool to assess sarcopenia. J. Gastrointest. Sur..

[26] Gallizzi M., Gagnon C., Harden R.N., Stanos S., Khan A. (2008). Medication Quantification Scale Version III: Internal validation of detriment weights using a chronic pain population. Pain Pract..

[27] Schizas C., Theumann N., Burn A., Tansey R., Wardlaw D., Smith F.W., Kulik G. (2010). Qualitative grading of severity of lumbar spinal stenosis based on the morphology of the dural sac on magnetic resonance images. Spine.

[28] Lee S., Lee J.W., Yeom J.S., Kim K.-J., Kim H.-J., Chung S.K., Kang H.S. (2010). A practical MRI grading system for lumbar foraminal stenosis. Am. J. Roentgenol..

[29] Naruishi K., Yumoto H., Kido J.-i. (2018). Clinical effects of low body mass index on geriatric status in elderly patients. Exp. Gerontol..

[30] Rosenberg I.H. (1997). Sarcopenia: Origins and clinical relevance. J. Nutr..

[31] Friedman J., Lussiez A., Sullivan J., Wang S., Englesbe M. (2015). Implications of sarcopenia in major surgery. Nutr. Clin. Pract..

[32] Beaudart C., Zaaria M., Pasleau F., Reginster J.-Y., Bruyère O. (2017). Health outcomes of sarcopenia: A systematic review and meta-analysis. PLoS ONE.

[33] Kaplan S.J., Pham T.N., Arbabi S., Gross J.A., Damodarasamy M., Bentov I., Taitsman L.A., Mitchell S.H., Reed M.J. (2017). Association of radiologic indicators of frailty with 1-year mortality in older trauma patients: Opportunistic screening for sarcopenia and osteopenia. JAMA Surg..

[34] Moisey L.L., Mourtzakis M., Cotton B.A., Premji T., Heyland D.K., Wade C.E., Bulger E., Kozar R.A. (2013). Skeletal muscle predicts ventilator-free days, ICU-free days, and mortality in elderly ICU patients. Crit. Care.

[35] Hawkins R.B., Mehaffey J.H., Charles E.J., Kern J.A., Lim D.S., Teman N.R., Ailawadi G. (2018). Psoas muscle size predicts risk-adjusted outcomes after surgical aortic valve replacement. Annal. Thorac. Sur..

[36] Weinstein J.N., Tosteson T.D., Lurie J.D., Tosteson A.N., Blood E., Hanscom B., Herkowitz H., Cammisa F., Albert T., Boden S.D. (2008). Surgical versus nonsurgical therapy for lumbar spinal stenosis. N. Engl. J. Med..

[37] Kobayashi S., Takeno K., Miyazaki T., Kubota M., Shimada S., Yayama T., Uchida K., Normura E., Mwaka E., Baba H. (2008). Effects of arterial ischemia and venous congestion on the lumbar nerve root in dogs. J. Orthop. Res..

[38] Chen J., Wang J., Wang B., Xu H., Lin S., Zhang H. (2016). Post-surgical functional recovery, lumbar lordosis, and range of motion associated with MR-detectable redundant nerve roots in lumbar spinal stenosis. Clin. Neurol. Neurosur..

[39] Moon D.E., Park H.J., Kim Y.H. (2015). Assessment of clinical outcomes of cervical epidural neuroplasty using a Racz-catheter and predictive factors of efficacy in patients with cervical spinal pain. Pain Physician.

[40] World Health Organization (1995). Physical Status: The Use of and Interpretation of Anthropometry, Report of a WHO Expert Committee.

[41] Abe T., Kearns C., Fukunaga T. (2003). Sex differences in whole body skeletal muscle mass measured by magnetic resonance imaging and its distribution in young Japanese adults. Br. J. Sports Med..

[42] Schorr M., Dichtel L.E., Gerweck A.V., Valera R.D., Torriani M., Miller K.K., Bredella M.A. (2018). Sex differences in body composition and association with cardiometabolic risk. Biol. Sex Differ..

